# Improved COOT optimization: An approach to multilevel thresholding in image segmentation

**DOI:** 10.1038/s41598-025-26283-8

**Published:** 2025-11-21

**Authors:** Simrandeep Singh, Harbinder Singh, Seyed Jaleleddin Mousavirad, Diego Oliva, Abdelazim G. Hussien, Laith Abualigah

**Affiliations:** 1 Department of Electronics & Communication Engineering, UCRD, Chandigarh University, Gharuan, Punjab India; 2 VISILAB, Universidad de Castilla-La Mancha, 13071 Ciudad Real, Spain; 3 Department of Computer and Electrical Engineering, Mid Sweden University, Sundsvall, Sweden; 4 Depto. Innovación, Basada en la Información y el Conocimiento, Universidad de Guadalajara, CUCEI, Av. Revolution, 1500 Guadalajara, Jal Mexico; 5 Department of Computer and Information Science, Linköping University, 581 83 Linköping, Sweden; 6 Computer Science Department, Al al-Bayt University, Mafraq, 25113 Jordan; 7 Hourani Center for Applied Scientific Research, Al-Ahliyya Amman University, Amman, 19328 Jordan

**Keywords:** Metaheuristics, Image segmentation, Multilevel thresholding, CT images, Computational science, Computer science

## Abstract

Image thresholding is one of the fastest and easiest approach for image segmentation and serves as a preprocessing step in computer vision and image processing applications, such as surveillance, image perception, scene understanding, artificial intelligence, augmented reality, biomedical imaging, remote sensing, image fusion, etc. Metaheuristic approaches have recently gained attention in the field of image segmentation. The standard COOT algorithm is a good alternative for solving complex optimization problems; however, it suffers from drawbacks, such as stagnation and insufficient balance between exploration and exploitation. This paper proposes the application of an improved COOT (ICOOT) optimization algorithm for multilevel image thresholding. In the proposed method, Lévy flights are incorporated to enhance the exploration capability of COOT, and quasi-opposition-based learning is introduced to improve the exploitation capacity and balance exploration and exploitation. To verify the efficiency of the ICOOT algorithm, it has been tested for solving complex optimization problems from the CEC’17 benchmark. The ICOOT algorithm is also employed to calculate the threshold values in image segmentation via Otsu’s entropy as an objective function for practical purposes. In addition to testing its performance in image thresholding, the proposed ICOOT algorithm has also been tested on benchmark images and computed tomography (CT) images from COVID-19 patients. The presented approach is compared with various state-of-the-art algorithms, and the ICOOT results outperform them.

## Introduction

Image segmentation plays a fundamental role in computer vision and medical imaging, enabling the separation of meaningful regions from complex visual data. It is an essential process in the fields of biomedical diagnosis, pattern recognition, and digital image processing^1^ and has been employed in a variety of applications, including ship target segmentation, optical character recognition (OCR), surveillance, industrial inspection, remote sensing, and biomedical research^2^. The purpose behind segmentation is to separate an image into homogenous groups, and these groups are called classes. The categorization into distinct classes is based on factors such as the grayscale intensity, color intensity, spatial texture, and geometric shape. Image segmentation plays a critical role in differentiating between healthy and cancerous tissues, including tumors. The most commonly preferred segmentation techniques available in the literature are clustering^3^, region^4^, graph, and thresholding^5^.

Among these, thresholding is most extensively adopted because of its superior efficiency and resilience compared with other techniques. Furthermore, thresholding can be categorized into bilevel and multilevel thresholding on the basis of the number of threshold values^6^. In bilevel thresholding, a unique threshold value is chosen to divide the image into two homogenous foreground and background portions on the basis of a gray histogram but it is insufficient for capturing the heterogeneity of image intensities. However, multilevel segmentation divides an image into several different areas via histogram values, which represent pixel intensities^7^. Bilevel thresholding, which employs a single global threshold, often produces results that are not sufficiently reliable for real-world applications, particularly when dealing with complex biomedical images. In such scenarios, multilevel thresholding is preferred for its ability to provide more nuanced segmentation. Choosing an optimal threshold value from the pool of random histogram values is a complex task. As a result, it is transformed into an optimization problem that may be resolved via parametric or nonparametric approaches. However, thess methods is computationally expensive and its complexity increases exponentially with the number of thresholds, making the process challenging for high-resolution and noisy images^8^.

Several metaheuristic optimization algorithms have been widely adopted for addressing these challenges. Consequently, numerous advanced techniques—such as multilevel thresholding, region growing, metaheuristic algorithms, artificial neural networks (ANNs), and clustering algorithms—are continuously evolving to enhance segmentation accuracy and efficiency^9^. Different classes may be specified in an image by employing probability density models in a parametric methodology; however, this strategy is computationally expensive. On the other hand, nonparametric techniques employ parameters to divide images into homogenous areas before thresholds are determined via quantifiable metrics (commonly Entropy or variance)^10^. To reach the optimal threshold values, several criteria have been presented by researchers, including Otsu’s between-class variance^11^, Renyi’s Entropy^12^, Kapur’s Entropy^13^, and Tsallis entropy^14^, among others. Otsu’s approach searches for the best between-class variance of each divided class, and Kapur’s method uses the histogram’s Entropy as a calculation to find the best threshold value.

Metaheuristic approaches such as swarm intelligence (SI)^15^ like Particle Swarm Optimization (PSO)^16^, Ant Colony Optimization (ACO)^17^, Artificial Bee Colony (ABC)^9^, are extensively used in the associated literature to address these difficulties on these grounds. Nature has inspired metaheuristic algorithms to find applications in many areas, such as physics, biology, and social behavior.

The COOT optimization algorithm was introduced as a metaheuristic approach inspired by the movement behavior of the Coot bird in nature^18^. Coot birds are water birds and exhibit distinctive decorated forehead and red dark eyes. This algorithm is inspired by the unique beahvioural pattern of these birds over the water surface. These birds demonstrate two unique movement patterns on the water surface: synchronous and disordered movement. Synchronized movement, where individuals follow a coordinated, chain-like formation across the water, and disordered movement, characterized by erratic and unstructured locomotion. From such basic movements, four operators for the COOT algorithm are extracted: 1) a random movement, 2) a chain movement, 3) adjusting the position on the basis of the group leader, and 4) a leader movement. By using such operators, the COOT method can explore and exploit the search space in an optimization process. Despite its strengths, it suffers from low convergence speed, limited diversity, and an imbalance between exploration and exploitation.

The primary contributions of this paper are as follows: a) An Improved COOT (ICOOT) algorithm is developed for addressing the image segmentation task, utilizing Otsu’s method as the objective function to determine optimal threshold values. b) A Lévy flight mechanism is incorporated to enhance the exploration capability of the standard COOT algorithm. c) A quasi opposition-based learning (QOBL) strategy is integrated to strengthen the exploitation capability. Furthermore, proposed ICOOT is compared with established existing state-of-the-art metaheuristic techniques, and its performance is quantitatively assessed.

The remainder of this paper is structured as: Section “Literature review” explains the literature review on multilevel thresholding and metaheuristic-based segmentation techniques. Section “Improved COOT (ICOOT) optimization algorithm” presents the Improved COOT (ICOOT) in detail. Section “Problem formulation via an ICOOT algorithm” formulates the segmentation problem within the ICOOT. Section “Discussion” describes an in-depth discussion of the proposed approach. Section “Experimental results” presents the results and analysis. Finally, conclusions, and future scope are explained in Section “Conclusion”.

## Literature review

Metaheuristic optimization techniques are effective computational tools for solving complex problems such as multilevel thresholding. This challenge has been addressed using both deterministic and metaheuristic approaches. Classical deterministic methods, such as Otsu’s between-class variance, Kapur’s Entropy, and Renyi’s Entropy, have been widely used due to their mathematical simplicity and interpretability. However, when increasing the threshold values these methods are not able to handle high-dimensional and highly multimodal search spaces, often become computationally expensive and struggle to produce optimal results.

Metaheuristic algorithms overcome these limitations by employing adaptive search mechanisms that balance exploration and exploitation, allowing efficient navigation of complex solution spaces. These algorithms are often inspired by natural phenomena and provide a set of structured rules and operators to guide the search process. Nature-inspired optimization algorithms are generally classified into two main categories: metaphor-based approaches, which derive inspiration from biological, physical, or social processes, and non-metaphor-based approaches, which are developed based on mathematical or heuristic principles without direct reference to natural analogies.

Metaphor-based algorithms follow principles derived from natural phenomena, mathematical concepts, or human social behaviors. These algorithms can be further divided into subgroups such as biology-based algorithms, enthused by biological processes and structures; chemistry-based algorithms, which replicate by chemical reactions and molecular interactions; physics-based algorithms, which are inspired by the laws of physics; mathematics-based algorithms; music-based approaches; and human-based algorithms, which imitate social behavior of human beings. Examples of human-based algorithms include the Heap-Based Optimizer (HBO), Teaching–Learning-Based Optimization (TLBO), and the League Championship Algorithm (LCA).

In recent years, numerous metaheuristic algorithms have been applied to multilevel thresholding, including swarm intelligence (SI)^15^ like Particle Swarm Optimization (PSO)^16^, which models the social behavior observed in flocking birds. In PSO, each particle in the swarm represents a potential solution to the optimization problem, with particles moving through the search space by adjusting their positions based on both their own experiences and those of neighboring particles. This cooperative mechanism enables the swarm to converge toward optimal or near-optimal solutions by striking a balance between exploration and exploitation. Each particle updates its position based on both its own best-found solution and the globally best solution identified within the swarm. Similarly, the Artificial Bee Colony (ABC)^9^ algorithm simulates the foraging behavior of bees to generate optimal solutions. At the same time, Ant Colony Optimization (ACO)^17^ is modeled on the pheromone-laying and path-following behavior of ants, enabling collective guidance toward optimal solutions.

In addition to PSO, several other bio-inspired algorithms have been developed based on such behaviors, such as the Grey Wolf Optimization (GWO) algorithm^19^, which draws inspiration from the social hierarchy and cooperative hunting strategy of grey wolves. Similarly, the Cuckoo Search (CS)^20^ algorithm is based on the brood parasitism behavior of cuckoos and incorporates random mutation mechanisms to explore the solution space. Simulated Annealing (SA), inspired by the annealing process in metallurgy, employs a heating and gradual cooling procedure to achieve a stable, low-energy crystalline structure. In optimization, it is particularly effective for navigating complex landscapes with numerous local optima in pursuit of the global optimum. The Whale Optimization Algorithm (WOA)^21^emulates the bubble-net feeding strategy of humpback whales, while the (AOA) relies on the mathematical principles of addition, subtraction, multiplication, and division to guide the search process^22^. More recently, the Coot Optimization Algorithm (COOT)^18^ has been introduced, which models the cooperative foraging behaviors and movement patterns of coot birds on water surfaces to address continuous optimization problems efficiently.

Many other such algorithms have also been Quantum Marine Predator Algorithm (QMPA), Hunger Games Search (HGS), Tunicate Swarm Algorithm (TSA), Teaching Learning Based Optimization (TLBO)^23^, Salp Swarm Algorithm (SSA)^24^, Pied Kingfisher Optimizer (PKO), and Bald Eagle Search (BES), etc. In addition to these optimizations, many modified versions of these algorithms have also been proposed, such as Cuckoo Search Algorithm via Lévy flights, Learning enthusiasm-based TLBO (LebTLBO)^1,25^, Modified Naked Mole Rat Optimization (mNMRO)^26,27^, and Slime Mould Algorithm (SMA)^28^.

Recent studies have introduced several improved or hybrid metaheuristic algorithms to address challenges in optimization and image segmentation tasks. Hu et al. proposed the Improved Hybrid Grey Wolf Optimizer with Seagull Optimization Algorithm (IGWO-SOA)^29,30^, which integrates the Grey Wolf Optimizer (GWO) with the Seagull Optimization Algorithm (SOA) to enhance the performance of K-means clustering for design engineering problems. Zhang et al. developed the Aptenodytes Forsteri Optimization Algorithm with Adaptive Perturbation of Oscillation and Mutation (AFOA-APM)^31^, inspired by the social behavior of emperor penguins, where an adaptive perturbation strategy and random mutation are employed to improve search efficiency.

Shi et al. introduced the Improved Whale Optimization Algorithm (CVWOA)^32^, which incorporates a convex local directed random search and a vortex rotation mechanism to refine whale optimization; the method was validated on the Berkeley and LN segmentation datasets. Similarly, Elaziz et al. proposed the Modified Planet Optimization Algorithm (MPOA)^33^, which enhances the original Planet Optimization Algorithm (POA) by integrating operators from the Reptile Search Algorithm (RSA), and applied it to polyp image segmentation. Adegboye et al. presented the Enhanced Exponential Distribution Optimizer (mEDO)^34^, which utilizes a phasor operator to enhance diversity and an adaptive mutation strategy to prevent local optima, demonstrating its effectiveness in machine learning parameter tuning.

Yu et al. proposed the Ensemble Grey Wolf Optimizer (EGWO), an enhanced version of GWO that incorporates an elite-based random search mechanism. They applied it successfully to image segmentation tasks^35^. More recently, Lakshmi et al. introduced the Slender Loris Optimization Algorithm (SLOA), which models the “climbing” (exploitation) and "jump-to" (exploration) behaviors of slender lorises to solve complex optimization problems effectively. The COOT algorithm and its modified versions have shown promising results in different applications, including hybrid scientific article recommendation^36^, image classification^37^, and waiting time prediction^38^.

Li et al. introduced an innovative Unsupervised Lévy-Flight with Particle Swarm Optimization (ULPSO) approach for image classification that achieves an effective balance between exploration and exploitation^39^. This method employs a search strategy in which, at every iteration, the position of the swarm’s worst-performing particle is updated using the Lévy Flight mechanism. LSHADE, combined with minimum cross-entropy, demonstrates superior performance in brain tumor detection compared to other algorithms^40^. The improved Chameleon Swarm Algorithm (ICSA), which leverages the Kapur and Otsu criteria, was applied for MRI-based disease identification, outperforming competitors in terms ofapur as a function^41^. Modified Whale Optimization Algorithm (mWOA) was applied to COVID-19 chest X-ray segmentation, providing faster computation^21^. Partitioned and Cooperative Quantum-behaved PSO (SCQPSO) applied Otsu’s Entropy for medical image segmentation, outperforming enhanced QPSO^39^. Adaptive Wind Driven Optimization (AWDO) segmented MRI brain images using both the Kapur and Otsu methods, achieving superior computation time and evaluation metrics^42^. Hybrid COVIDOA-HHO algorithm was proposed for 2D and 3D medical image segmentation, surpassing several competing algorithms^43^. The Coronavirus Disease Optimization Algorithm (COVIDOA) was applied for skin cancer detection, demonstrating competitive performance using Otsu, Kapur, and Tsallis thresholding^43^. The Enhanced Differential Evolution Algorithm was used for breast cancer segmentation, indicating high segmentation quality^44^. Furthermore, LCWOA, integrating a Lévy operator and chaotic random mutation into WOA, was employed for skin cancer detection, providing improved segmentation results over baseline methods^21^. Recent studies have explored various metaheuristic strategies for multilevel thresholding in image segmentation. The Black Widow Optimization (BWO) algorithm has been applied to this problem with Otsu and Kapur entropy functions serving as the objective criteria^45^. Learning Enthusiasm-Based Teaching–Learning-Based Optimization (LebTLBO), recognized for its simplicity and low computational overhead, has likewise demonstrated effectiveness^46^.

Hybrid approaches combining different metaheuristics have also gained attention. For example, a cuckoo search–differential evolution (DE) hybrid integrates the mutation operator of DE to enhance cuckoo search performance^47^, while a dragonfly algorithm (DA) coupled with DE employs DE as a local search mechanism^48^. A self-adaptive DE has been merged with the grasshopper optimization algorithm (GOA)^49^, allowing dynamic switching between the two based on the population’s average fitness. In breast cancer image segmentation, the slime mould algorithm (SMA) has been used as a post-processing step following DE, optimizing a two-dimensional Kapur entropy objective^50^. Other hybrids include a DE–Harris Hawks Optimization (HHO) scheme for 2-D Masi entropy-based thresholding^51^, and a method that blends cuckoo search with biogeography-based optimization (BBO) by incorporating a BBO discovery operator into a heterogeneous cuckoo search framework^52^.

In this study, we propose the ICOOT optimization algorithm for multilevel thresholding of images. The COOT algorithm has demonstrated strong potential in addressing numerical optimization benchmark problems and various engineering applications^18,53^. Nevertheless, it exhibits certain limitations, including relatively slow convergence, a tendency to stagnate in local optima, inadequate balance between exploration and exploitation, and limited population diversity^54^. To overcome these shortcomings, this study introduces an enhanced variant, termed Improved COOT (ICOOT), specifically designed to mitigate these issues and improve overall optimization performance. The following two stages are employed in the ICOOT version: 1) Lévy flights^55^ and 2) Quasi Opposition-based Learning (QOBL)^56^. Lévy flights are amended to increase the exploration ability of the basic COOT algorithm. However, quasi opposition-based Learning is presented to improve the exploitation capacity. The presented algorithm creates a balance between exploration and exploitation and prevents it from becoming stuck in local solutions. The suggested ICOOT algorithm is used in the image segmentation process.

## Improved COOT (ICOOT) optimization algorithm

### COOT optimization algorithm

**Fig. 1:**
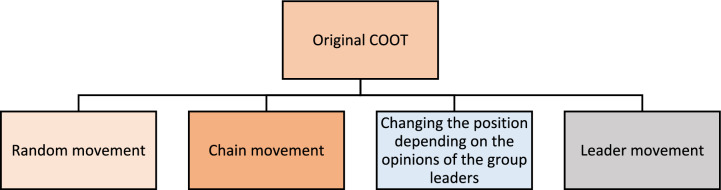
Different phases of the original COOT algorithm.

The practice of identifying a solution or global optimum solution for a challenge is known as optimization. The global optimum value is calculated by maximizing or minimizing the objective function or fitness value. Optimization challenges may be addressed across all sectors of engineering and science. Hence, this field has become a significant and necessary area of research for scholars. Metaheuristic algorithms may be seen as a type of random method that determines the best answer. Naruei et al. presented a novel optimization scheme inspired by the COOT bird natural life model^18^. The behavior of COOTs (water birds) on water is considered the basis of this algorithm. The basic drawback of optimization is that it traps local minima with a slow convergence rate, resulting in an imbalance between exploration and exploitation. It has four phases, as described in Fig. 1.

### Mathematical model

COOT optimization starts with the initialization of a random variable set. Optimization algorithms depend upon random variable results; however, they do not guarantee that every result is the same. Although the same equation is followed for several iterations, the probability of finding the best solution increases many-fold. The random population is generated as follows Eq. 1^18^:

1 $$CootPos\left(i\right)=rand\left(1,d\right).*\left(ub-lb\right)+lb$$

where $CootPos$ represents the bird’s position, *d* is the problem dimension, and *ub* and *lb* are the upper bound and lower bound values, respectively. The four different phases may be mathematically modelled as follows. The first phase is the random movement of birds in different directions described by the random position R, which is defined by Eq. 2^18^.

2 $$R=rand\left(1,d\right).\times \left(ub-lb\right)+lb$$

To escape the solution from becoming stuck in local minima, the new position of COOT is defined by Eq. 3^18^.

3 $$CootPos\left(i\right)=CootPos\left(i\right)+B\times {R}_{1}\times (R-cootPos\left(i\right))$$

where *B* is defined in Eq. (4), and a new variable ${R}_{1}$ is introduced, which is a random number between [0, 1].

4 $$B=1-{I}_{c}\times \frac{1}{{I}_{max}}$$

where ${I}_{c}$ is the current iteration and where ${I}_{max}$ represents the maximum number of iterations.

### Chain movement

The chain movement is the regular drive of COOTs one after the other in a chain manner, and it may be calculated by taking the average position of two birds as described by eq^18^..

5 $$CootPos\left(i\right)=\frac{CootPos\left(i\right)+CootPos\left(i-1\right)}{2}$$

where *(i)* is the COOT position at any given instant and where $(i-1)$ is the position behind a given COOT.

The learner COOTs divert their location and follow the path shown by the group leaders. The position of a given COOT is defined by the following eq. depending on the opinions of the group leaders:

6 $$K = 1 + (jMOD N{I}_{c})$$

where *j* represents the count of the current COOT and N ${I}_{c}$ describes the total leader number. The *K* label is used to indicate the leader number and helps to define the position of the next COOT according to:

7 $$CootPos\left(i\right)=LeaderPos\left(k\right)+2\times {R}_{2}\times \text{cos}(2\times \pi \times {R}_{3})\times (LeaderPos\left(k\right)-CootPos(i)$$

where *R*_*3*_ is a random number in the range of [−1, 1].

Every group leader of the COOT group will find the optimal global position and continue updating their current position continuously, according to Eq. 8.

8 $$LeaderPos\left(i\right)=\left\{\begin{array}{c}B\times {R}_{4}\times \text{cos}\left(2\pi {R}_{3}\right)\times \left(gBest-LeaderPos\left(i\right)\right)+gBest {R}_{5}<0.5\\ B\times {R}_{4}\times \text{cos}\left(2\pi {R}_{3}\right)\times \left(gBest-LeaderPos\left(i\right)\right)-gBest {R}_{5}\ge 0.5\end{array}\right\}$$

where $gBest$ is the global best or optimal position and where ${R}_{4} \&$ ${R}_{5}$ are random numbers ranging from [0, 1].

### ICOOT algorithm

The Improved COOT (ICOOT) algorithm integrates the Lévy flight mechanism to significantly enhance the exploration capability of the standard COOT algorithm by allowing the search agents to perform long-distance moves, thereby increasing the likelihood of escaping local optima and covering a wider search space. To complement this, the exploitation ability is strengthened to ensure that the algorithm can effectively refine solutions in promising regions. A crucial aspect of ICOOT is maintaining a proper balance between exploration and exploitation, which is achieved through the combined use of Lévy flight and quasi-opposition-based Learning (QOBL). The QOBL component introduces diverse candidate solutions by considering their opposite positions in the search space, thus improving population diversity and accelerating convergence toward the global optimum. This synergistic combination of exploration, exploitation, and diversity preservation enables ICOOT to deliver more accurate and robust performance across complex optimization tasks.

#### Lévy flights

Lévy flights are inherently Markov processes, representing a type of random walk^55^ characterized by step lengths that follow a Lévy distribution. In the ICOOT framework, the Lévy flight distribution is incorporated to reduce the likelihood of algorithmic stagnation and avoid entrapping in local optima. This mechanism enhances the exploration strength and potential of the optimizer by increasing the probability of generating diverse and novel solutions. Essentially, Lévy flight is a stochastic procedure that generates new candidate solutions through a random walk governed by Lévy steps. Eq. (9) describes the updated population positions derived from the Lévy distribution^28^.

9 $${CootPos\left(i\right)}^{new}= CootPos\left(i\right)\oplus \propto \times Levy (\beta )$$

where $\propto$ corresponds to an irregular step size, $\oplus$ denotes entrywise multiplication, and $\beta$ is a Lévy Flight distribution parameter. The Lévy flight can be described as follows:

10 $$Levy=0.01 \times \frac{{R}_{6}\times \sigma }{{\left|{R}_{7}\right|}^{ \frac{1}{\beta }}}$$

11 $$\sigma ={\left(\frac{\Gamma (1+\beta )\times \text{sin}\left( \frac{\pi \beta }{2}\right)}{\Gamma \left(\frac{1+\beta }{2}\right)\times \beta \times 2\left( \frac{\beta -1}{2}\right)}\right)}^{\frac{1}{\beta }}$$

where ${R}_{6}$ and ${R}_{7}$ are arbitrary values taken in the limit [0,1], and β is assumed to be 1.5.

#### Opposition-based learning

Tizhoosh introduced Opposition-Based Learning (OBL)^57^ as an effective mechanism to enhance optimization efficiency and accelerate convergence. The fundamental principle of OBL is to simultaneously consider a candidate solution and its opposite within the defined search space (see Eq. 12). Both the original and opposite solutions are evaluated using the objective function, and the one with better fitness is selected for progression to the next iteration. By integrating the concept of "opposite points," OBL effectively broadens the search coverage in fewer iterations, reducing the risk of getting trapped in local optima and improving the likelihood of reaching the global optimum. This strategy is particularly advantageous in the early stages of the search process, where it enables faster exploration, as well as in later stages, where it refines the search toward high-quality solutions. Compared to purely random initialization or movement, OBL tends to produce solutions that are statistically closer to the optimal region, thereby improving both search quality and convergence speed.

12 $${x}_{obl, i}\left(t\right)=ub+lb-{x}_{i}\left(t\right) , i \in [1, 2,\dots \dots \dots .., n]$$

where ${x}_{obl, i}\left(t\right)$ is the opposite solution, $ub$ and $lb$ are upper and lower bound respectively for current solution ${x}_{i}\left(t\right)$ at time *t*.

#### Quasi-opposition-based learning

Rahnamayan et al.^58^ introduced an enhanced variant of OBL known as Quasi-Opposition-Based Learning (QOBL), which keeps the basic mechanism same as that of original OBL approach but introduces a key variation. Standard OBL, which considers the exact opposite of a candidate solution, QOBL generates a quasi-opposite solution—an alternative that lies between the current solution and its true opposite (see Eq. 13). This subtle modification increases the possibility of locating optimal value that are near to the global optimum solution.

**Fig. 2:**
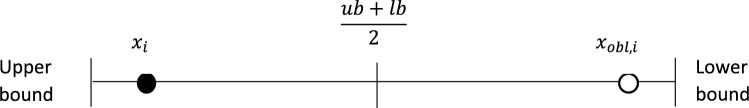
Quasi-opposition-based learning.

The quasi-opposite solution is computed based on the mathematical framework of opposition but adjusted to reflect an intermediate position, thereby combining the advantages of exploration and exploitation more effectively. Empirical evidence has shown that QOBL often surpasses conventional OBL in both convergence rate and solution accuracy, making it a preferred choice for many modern optimization algorithms aiming for faster and more reliable performance.

13 $${x}_{qobl, i}\left(t\right)=\text{rand}\left( \frac{ub+lb}{2}, {x}_{obl, i}\left(t\right)\right)$$

where ${x}_{obl, i}\left(t\right)$ represents the opposite solution, ${x}_{qobl, i}\left(t\right)$ denotes the quasi-opposite solution, $ub$ and $lb$ are the upper and lower bound respectively. The conceptual framework and representation of the Quasi-Opposition-Based Learning (QOBL) is illustrated in Fig. 2. The complete workflow of the proposed ICOOT approach, including initialization, position updates, and enhanced search strategies, is outlined in **Algorithm 1**.

**Algorithm 1:**
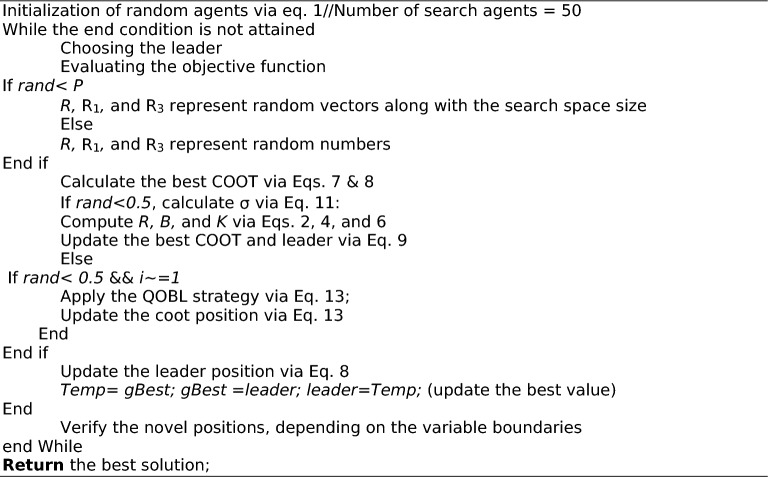
Pseudocode of the proposed ICOOT algorithm

## Problem formulation via an ICOOT algorithm

Image segmentation by thresholding techniques can be conducted via various methods that leverage information theory and probability distributions, such as Otsu’s between-class variance, Kapur’s Entropy^59^, Renyi’s Entropy^14^, and Tsallis entropy^17^. This paper uses Otsu’s between-class variance^60^ as the objective function, emphasizing its effectiveness. The 1-D Otsu method remains unaffected by the brightness and contrast of an image and boasts computational efficiency for a limited number of thresholds^6–9,23^. Although Otsu’s approach yields superior segmentation results, it is constrained by its exclusive consideration of gray level values, leading to suboptimal performance in the presence of image noise. The thresholding process, depending on the number of thresholds, can be characterized as bilevel (BT) or multilevel (MT) thresholding. Bilevel thresholding segregates an image into two distinct regions, whereas MT thresholding creates multiple regions within an image. The segmentation can be succinctly expressed via Eq. 14.

14 $$b(x,y)=\left\{\begin{array}{c}{a}_{1}\leftarrow b(x,y) if 0\le b(x,y)<Thresh\\ { a}_{2}\leftarrow b(x,y)if Thresh\le b(x,y)<L-1\end{array}\right\}$$

where $b(x,y)$ is an image of size $\left(m\times n\right)$ with *L* intensity levels and where ${a}_{1}$ and ${a}_{2}$ are two distinct classes that depend on the threshold value T $hresh.$ Sometimes, bilevel thresholding leads to an ambiguous threshold value (th), and in such cases, the MT is employed to enhance the segmentation results^8^. The distinct classes generated in the MT via multiple thresholds are given as ${a}_{1},{a}_{2},\dots .,{a}_{n}.$ The probability distribution ${p}_{i}$, which is mathematically defined as:

15 $${p}_{i}=\frac{{n}_{i}}{N}$$

where ${n}_{i}$ is the number of pixels with gray level *i* and *N* represents total number of pixels. The average gray level of an image *I* can be computed as:

16 $${\mu }_{I}=\sum_{i=0}^{L-1}{iP}_{i}$$

17 $${a}_{1}=\frac{{P}_{0}}{{\omega }_{a}}, \frac{{P}_{1}}{{\omega }_{a}},\frac{{P}_{2}}{{\omega }_{a}}\dots \dots \frac{{P}_{T}}{{\omega }_{a}}$$

18 $${a}_{2}=\frac{{P}_{T+1}}{{\omega }_{b}},\frac{{P}_{T+2}}{{\omega }_{b}}\dots \dots \frac{{P}_{L-1}}{{\omega }_{b}}$$

Where $L$ is the total number of possible gray levels in the image, $i$ denotes a particular gray level, and ${P}_{i}$ is probability distribution, where ${\omega }_{a}$ and ${\omega }_{b}$ denote the probabilities (class weights) of the respective classes ${\omega }_{a}= \sum_{i=0}^{T}{p}_{i} , {\omega }_{b}= \sum_{i=T+1}^{L-1}{p}_{i}$

The average gray levels ${\mu }_{a}$ and ${\mu }_{b}$ for the two classes, ${a}_{1}$ and ${a}_{2}$​ are computed as:

19 $${\mu }_{a }(k)=\sum_{i=0}^{T}\frac{{ip}_{i}}{{\omega }_{a}}, {\mu }_{b}(k)=\sum_{i=T+1}^{L-1}\frac{{ip}_{i}}{{\omega }_{b}}$$

If the mean intensity of the image is represented as ${\mu }_{I}$, then the relationship between the class means and their probabilities can be expressed as:

20 $${\omega }_{a}{\mu }_{a}+{\omega }_{b}{\mu }_{b}={\mu }_{I}, {\omega }_{a}+{\omega }_{b}=1$$

To determine the optimal threshold value, the between-class variance ${\sigma }_{B}^{2}$ is maximized using the following equation:

21 $${\sigma }_{B}^{2} ={\omega }_{a}{\left({\mu }_{a}-{\mu }_{I}\right)}^{2}+{\omega }_{b}{\left({ \mu }_{b}-{\mu }_{I}\right)}^{2}$$

A further extension from bilevel to multilevel thresholding may be carried out for the Otsu algorithm via the following rules.

$${a}_{1}\leftarrow b(x,y) if 0<b(x,y)<{T}_{1}$$

$${a}_{2}\leftarrow b(x,y) if {T}_{1}<b(x,y)<{T}_{2}$$

$${a}_{3}\leftarrow b(x,y) if {T}_{2}<b(x,y)<{T}_{3}$$

$${a}_{i}\leftarrow b\left(x,y\right)if {T}_{i-1}<b\left(x,y\right)<{T}_{i+1}\vdots$$

22 $${a}_{n}\leftarrow b(x,y) if {T}_{n-1}<b(x,y)<L-1$$

where *n* defines the number of classes such as [${a}_{1}, {a}_{2}, {a}_{3}, {a}_{i},\dots \dots ..{a}_{n}$] considering *i* as a certain class for a given image $b(x,y)$ having *L* gray levels (1,2,…, *L*) in the range [0, *L*−1]. The extension between the class values is given by the $f\left(k\right)$ between-class variance.

23 $$f\left(k\right)=\sum_{i=1}^{M}{\omega }_{i}{\left({\mu }_{i}-{\mu }_{T}\right)}^{2}$$

Otsu method for multilevel thresholding considering *M-1* thresholding levels is given by the following equation for *k* thresholds^2^.

24 $$f(k) ={\omega }_{a}(\text{k}){{\mu }_{a}}^{2}(k)+{\omega }_{b}(k){{\mu }_{b}}^{2}(k)$$

where ${\mu }_{a}$ and ${\mu }_{b}$ are average levels for classes ${a}_{1}$ and ${a}_{2}$ already explained in Eq. 19.

25 $${f}_{OTSU}(T)={\rm{\varnothing }}_{o}=\rm{Arg max}\left(f\left(k\right)\right), 0\le k\le L-1$$

Where ${f}_{OTSU}$ is an objective, and the required optimal threshold value of pixel can be derived from it by maximizing Eq. 25. Fitness function considering *i* multilevel threshold values is given by Eq. 26.

26 $${f}_{OTSU}({T}_{i})={\rm{\varnothing }}_{o}=\rm{Arg max}\left(f\left({k}_{i}\right)\right), 0\le k\le L-1, i=1, 2, \dots \dots ..,\rm{ T}$$

## Discussion

**Fig. 3:**
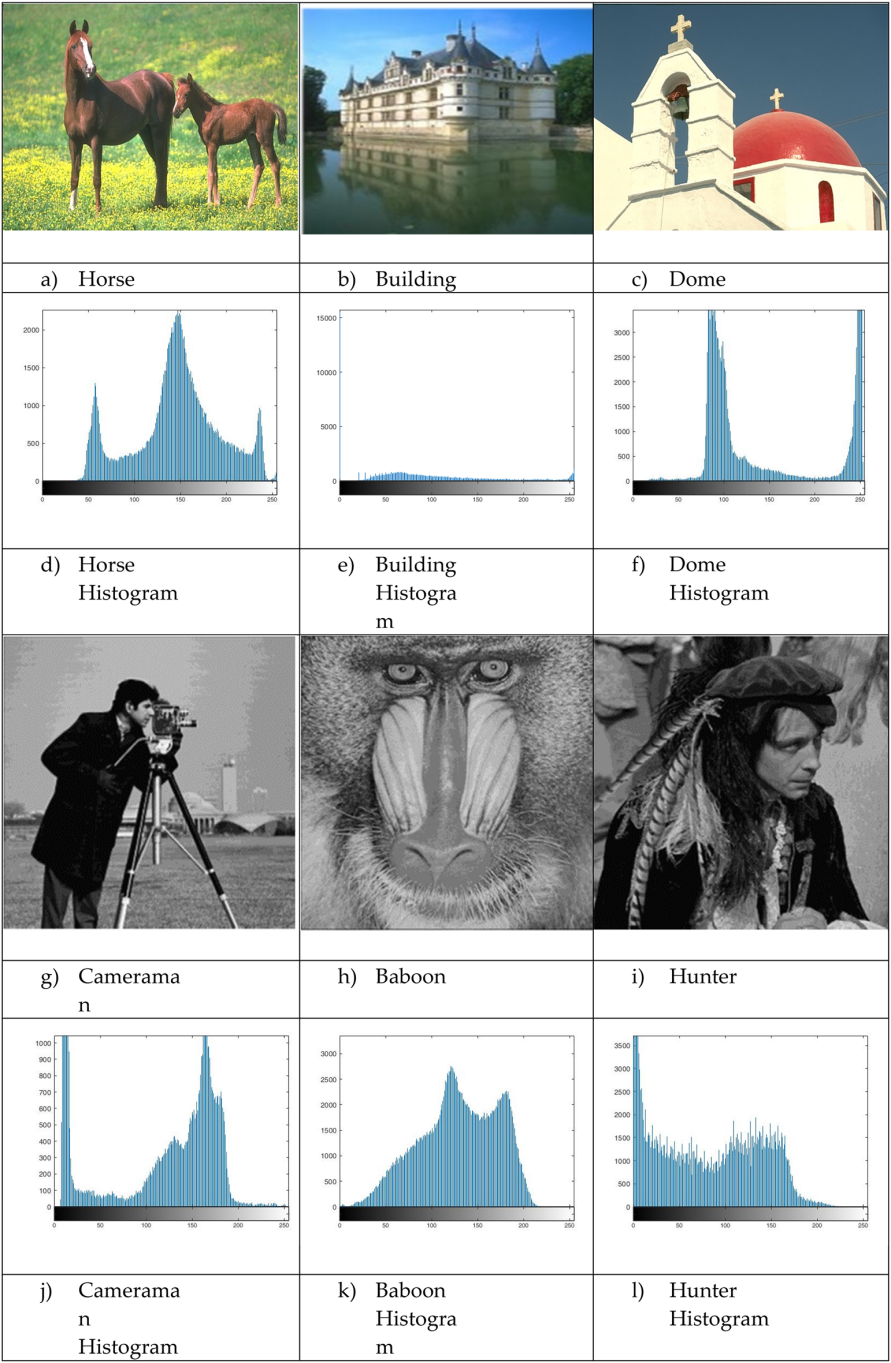

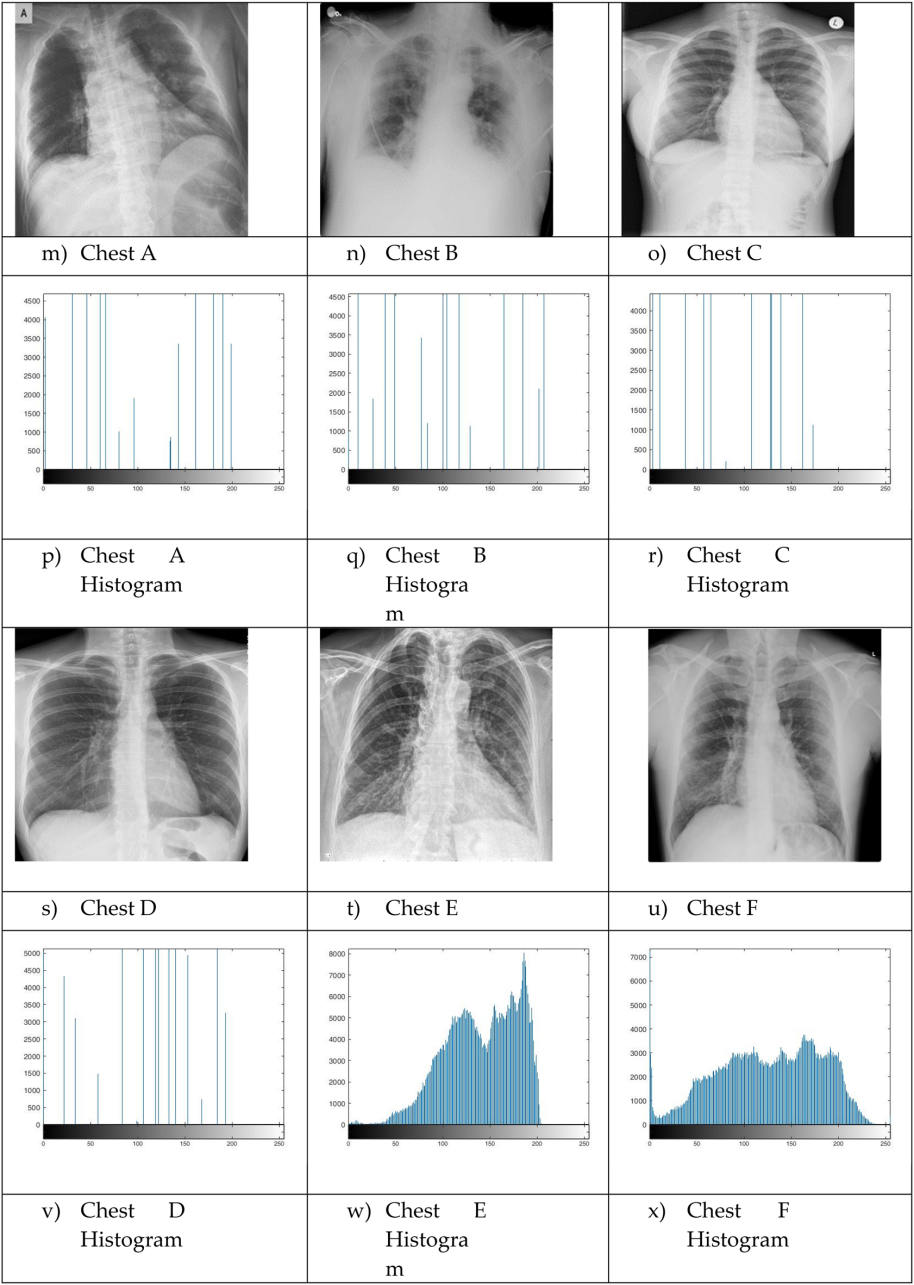
Benchmark images and corresponding histograms.

The proposed Improved COOT (ICOOT) algorithm is designed to address key limitations of the original COOT optimizer, namely its relatively slow convergence speed, tendency to stagnate in local optima, and imbalance between exploration and exploitation. By introducing adaptive control parameters and enhanced position update strategies, ICOOT aims to maintain a diverse population throughout the optimization process while accelerating convergence towards optimal solutions. The standard six benchmark images (Horse, Building, Dome, Cameraman, Baboon, and Hunter) and six COVID-19 images shown in Fig. 3 are chosen to showcase the presented approach. These images are chosen to prove the diversity of the presented approach, and they contain different patterns and shapes of histograms. Figure Horse, dome, Cameraman, and Chest D are the most crucial and critical images, as they contain multiple peaks. The proposed algorithms are executed in MATLAB 2021 via an Intel(R) Core(TM) i7-3520 M CPU @ 2.90 GHz GB RAM machine. To ensure a balanced and meaningful performance comparison with existing state-of-the-art multilevel thresholding techniques, a comparative analysis was carried using Aquila optimizer (AO)^63^, whale optimization algorithm (WOA)^64^, salp swarm algorithm (SSA)^24^, arithmetic optimization algorithm (AOA)^65^, particle swarm optimization (PSO)^16^, quantum marine predator algorithm (QMPA)^66^, and COOT optimization algorithm^18^. These optimization algorithms are widely recognized in the literature for their robustness and adaptability in solving complex multimodal problems, particularly in image processing and segmentation. They represent distinct conceptual paradigms: swarm intelligence (PSO, WOA, SSA, COOT), arithmetic-based search strategies (AOA), and predator–prey behavioural models (AO, QMPA).

## Experimental results

For a better performance evaluation of the ICOOT method, this paper uses more challenging optimization problems from the CEC’17 test suite^67^. The test bed consists of 30 functions, with functions F1 and F2 being eliminated^68^; thus, 28 functions of various modalities and difficulties are used to evaluate ICOOT as well as other equivalent methods chosen in this study. F3 is a unimodal function, F4--F10 is a subset of multimodal functions, F11--F20 correspond to hybrid functions, and the F21--F30 subset provides composition functions. All of the benchmarks preserve a [−100, 100] D hyperspace. Generally, CEC’17 is rather complicated and dynamic, allowing for in-depth investigation of a metaheuristic algorithm’s exploration and exploitation characteristics. This research conducts a statistical test to ensure that the experimental results are not coincidental and that the statistical performance between the ICOOT and other state-of-the-art approaches is satisfactory when various benchmark functions given by CEC’2017 are utilized. Table 1 shows that the ICOOT outperforms competing methods in the majority of benchmark functions and has fast convergence efficiency.

**Table 1:** The means and standard deviations of the fitness values produced by the rival methods over the CEC’2017 functions with Dim=30 (Iterations executed:2000).

**Function name**	**Measure**	**AO**	**WOA**	**SSA**	**AOA**	**PSO**	**QMPA**	**COOT**	**ICOOT**
F3	Mean	6470	1640	900	**300**	11820	2635	1360	910
	STD	64.4	20.3	20.1	15.2	3655	30.4	24.5	**5.6**
F4	Mean	6760	4780	8570	6250	691	4583	493	**469**
	STD	43.72	52.6	69.2	77.1	**3.7**	65.4	25.3	**3.7**
F5	Mean	638	874	822	839	795	683	625	**180**
	STD	33	66.4	66.9	73.7	69	**12.3**	35.4	22
F6	Mean	690	526	**400**	552	664	445	9540	600
	STD	4.21	2	2	4.9	**1.2**	14	18	1.9
F7	Mean	926	712	627	783	961	621	924	**614**
	STD	20	20	64	65	**19**	23	20	72
F8	Mean	668	642	**600**	668	1020	626	684	690
	STD	6.2	5.3	**0**	10	27	8	5	**0**
F9	Mean	3700	3110	2760	3690	4820	2448	2520	**1796**
	STD	**19.1**	105	30	37	172	22.4	25.9	46.6
F10	Mean	2560	2770	2230	2550	6670	**2030**	3050	**2030**
	STD	181	299	173	242	571	**145**	196	165
F11	Mean	1050	943	907	1010	1340	891	1180	**887**
	STD	**13**	16	73	59	49	19	19	86
F12	Mean	17,69,500	30,39,600	2,35,590	56,10,500	3670000	345000	510000	**130600**
	STD	8,21,520	19,79,500	1,37,000	33,63,900	16100000	31343	122000	**6000**
F13	Mean	**3,215**	6,026	8,089	35,581	8540000	9812	7400	7170
	STD	1,356	4,848	5,401	9,062	2870000	921	935	**128**
F14	Mean	1,489	1,504	4,957	32000	70600	123000	1489	**1470**
	STD	14	21	3,294	27500	46300	91000	18	13
F15	Mean	**54700**	108000	84600	62900	70600	83430	924000	151000
	STD	49500	82500	63200	59100	34400	13200	1480000	**10400**
F16	Mean	2820	3490	3700	3680	3090	**2690**	**2690**	3110
	STD	277	650	466	529	392	**188**	291	326
F17	Mean	3310	3170	2850	3180	**2430**	2950	3460	3920
	STD	70	62	52.7	83	204	32	51	**14**
F18	Mean	3330	2920	**2880**	2960	568000	2910	6160	**2880**
	STD	74	15	**0**	27	450000	16	14	469
F19	Mean	7150	4620	**4250**	7850	3390000	4610	9590	9660
	STD	643	1950	728	981	1710000	1410	**534**	1010
F20	Mean	3200	4080	3200	3360	**2690**	3270	3810	3240
	STD	**0**	357	**0**	99	168	3	75	**0**
F21	Mean	3980	4040	9780	7110	2550	**2300**	7960	**2300**
	STD	3090	3480	2680	3080	**38**	2670	3260	3790
F22	Mean	4160	2340	3080	1410	5950	1410	4850	**1390**
	STD	55	142	50.8	95	2810	37	54	**27**
F23	Mean	3200	3300	3430	3080	2810	**2790**	2830	3120
	STD	143	130	168	110	**14**	78.9	38	110
F24	Mean	2600	3020	2490	2770	3220	**2420**	2850	**2420**
	STD	88	235	136	194	98	113	**85**	184
F25	Mean	2570	2520	2400	2580	2930	2395	2680	**2380**
	STD	23	26	70	53	27	22	19	**17**
F26	Mean	728	534	1950	981	6200	1240	1430	**500**
	STD	3200	3810	4080	3360	**2170**	2340	3250	3120
F27	Mean	**3200**	3810	4080	3360	3250	3390	3250	3240
	STD	50.6	177	174	146	47	**2.87**	28.6	12
F28	Mean	3300	6270	3240	3310	3260	3150	3220	**3100**
	STD	**0.000092**	428	51.4	36	25	22.1	22	56.8
F29	Mean	4070	5610	4460	4940	4460	4480	3910	**3730**
	STD	**177**	270	230	339	251	341	245	200
F30	Mean	1340000	6300000	1920000	7160000	**160000**	345000	1150000	246720
	STD	964000	4070000	1690000	8270000	**151000**	490000	3460000	62579

The outcomes generated by optimization algorithms lack stability because of their reliance on stochastic methods and random numbers for essential parameters. To assess the robustness of the proposed techniques, the algorithms are tested 50 times across various threshold values ($Th=2, 3, 4, and 5$). The evaluation of thresholded images involves quantitative analysis via metrics such as the peak signal-to-noise ratio (PSNR), structural similarity index (SSIM), and feature similarity index (FSIM). The subsequent subsection provides a concise discussion of these parameters.

### Dataset

The ICOOT algorithm is applied and evaluated on the USC-SIPI image database^69^, a publicly available collection of over 400 digital images organized into four categories: textured, miscellaneous, aerial, and sequences. The database includes images in various resolutions, such as 256x256, 512x512, or 1024x1024 pixels. Black-and-white images are stored at 8 bits/pixel, while colour images are stored at 24 bits/pixel. For demonstration of this study, we have selected six digital benchmark images from miscellaneous category: Horse, Building, Dome, Cameraman, Baboon, and Hunter, each resized to 512 × 512 pixels to ensure uniformity across experiments.

To further examine the general capability of the algorithm, it is also tested on a different set of images taken from the Berkeley Segmentation Data^70^. This dataset is a widely used benchmark in image segmentation research and contains 500 natural colour images. Each image is accompanied by multiple human-labelled ground truth segmentations, which make it highly suitable for objective performance evaluation. In this study experiments were conducted on a wide range of medical CT images comprising scans of COVID-19 infected and normal patients. However, for demonstration purposes and to adhere to space constraints, results for six representative cases are presented in this paper, Specifically, Chest A, Chest B, Chest D, Chest E, and Chest F represents infected persons, while Chest C, correspond a normal case. All images were converted to grayscale and resized to 512 × 512 pixels to maintain uniform resolution across all experiments. The performance of the ICOOT algorithm is assessed by comparing the peak signal-to-noise ratio (PSNR) and structural similarity index measure (SSIM)^71^ with those of the aforementioned techniques.

The results for different threshold values are comprehensively presented in tabular format. Table 2 lists the outcomes of thresholding at the 2^nd^, 3^rd^, 4^th^, and 5^th^ levels. It includes the corresponding threshold values and the resulting data after applying these thresholds. Additionally, the table provides a convergence graph that visually represents how the algorithm’s performance stabilizes as the threshold values are adjusted across the different levels.

**Table 2:**
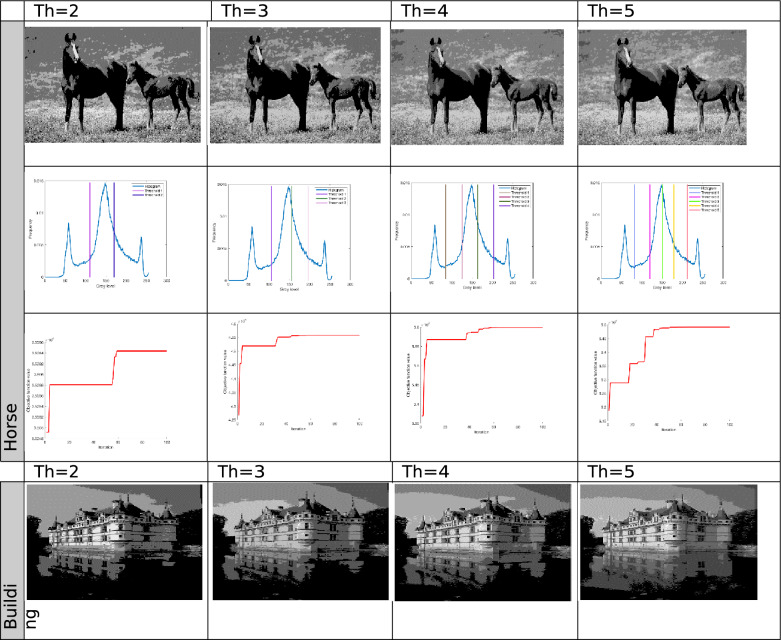

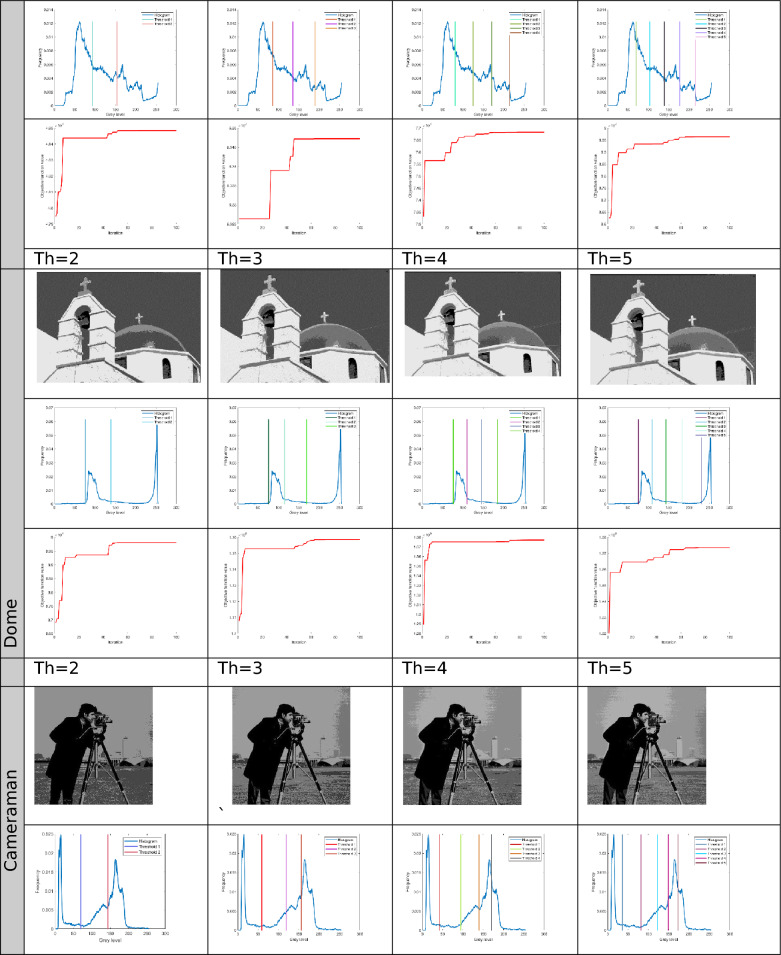

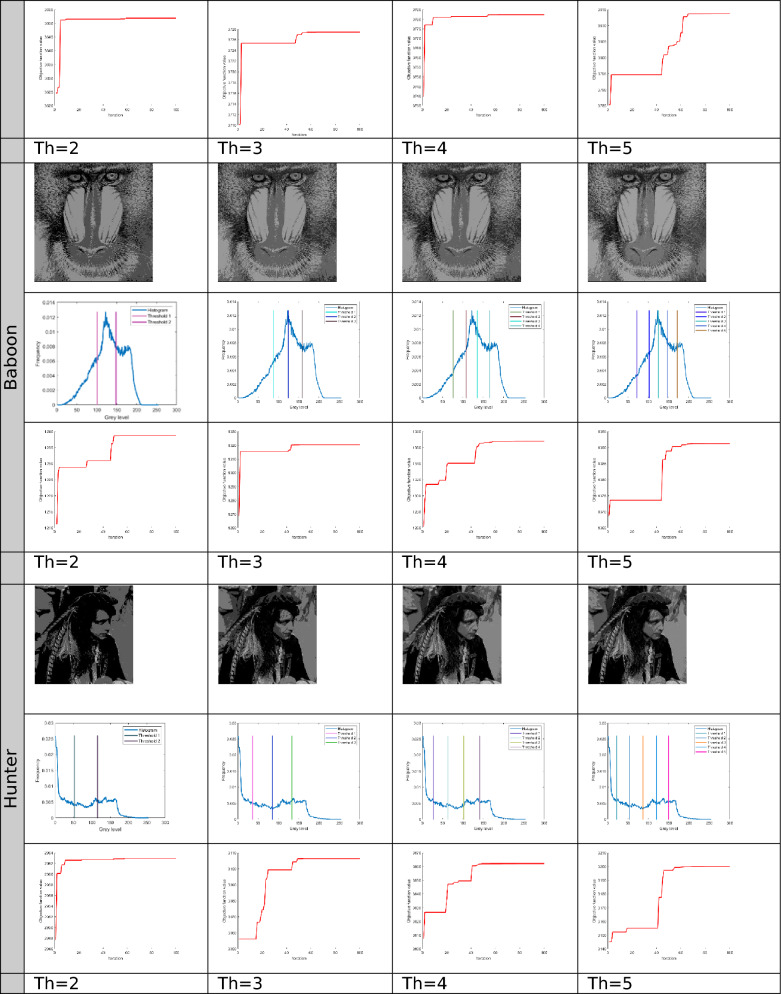

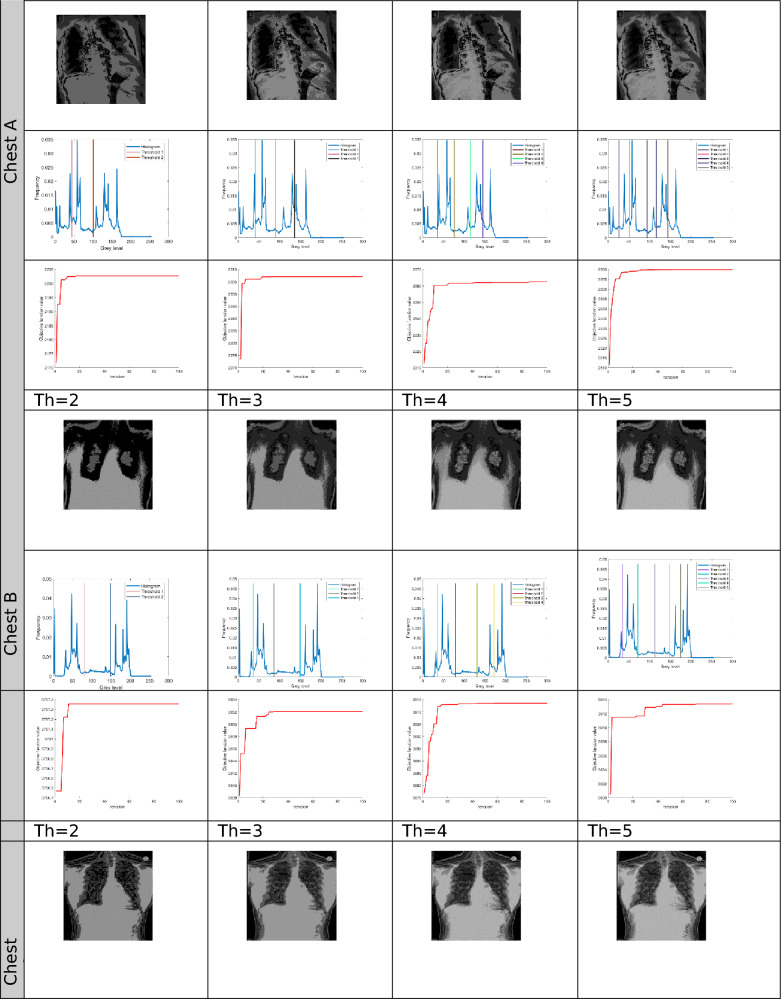

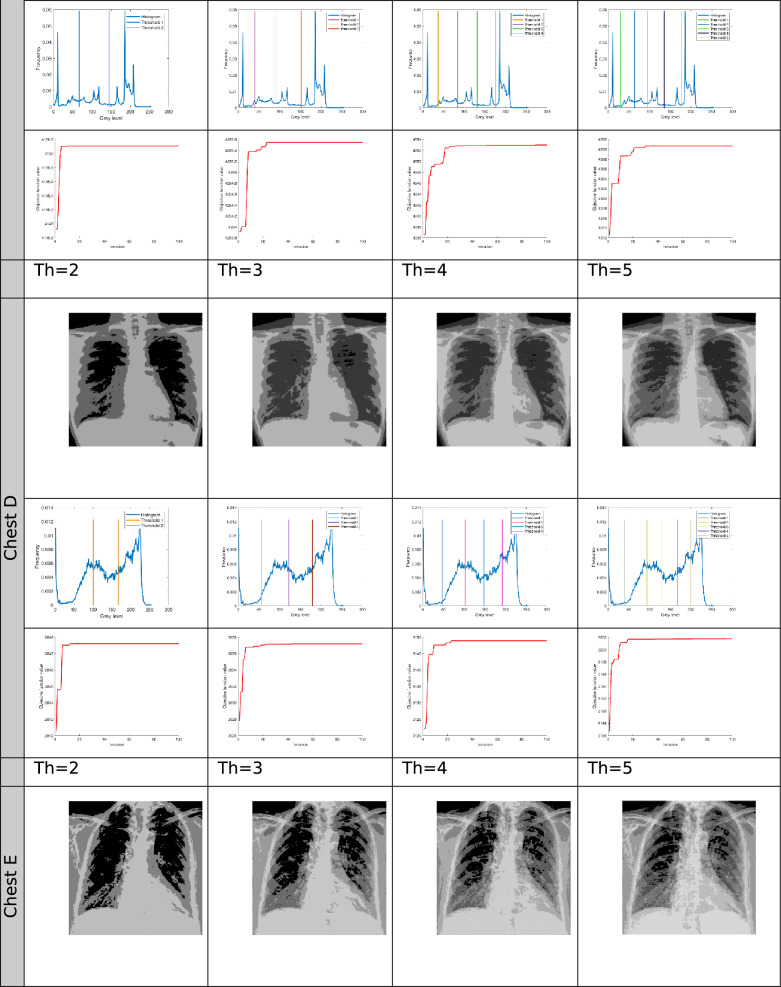

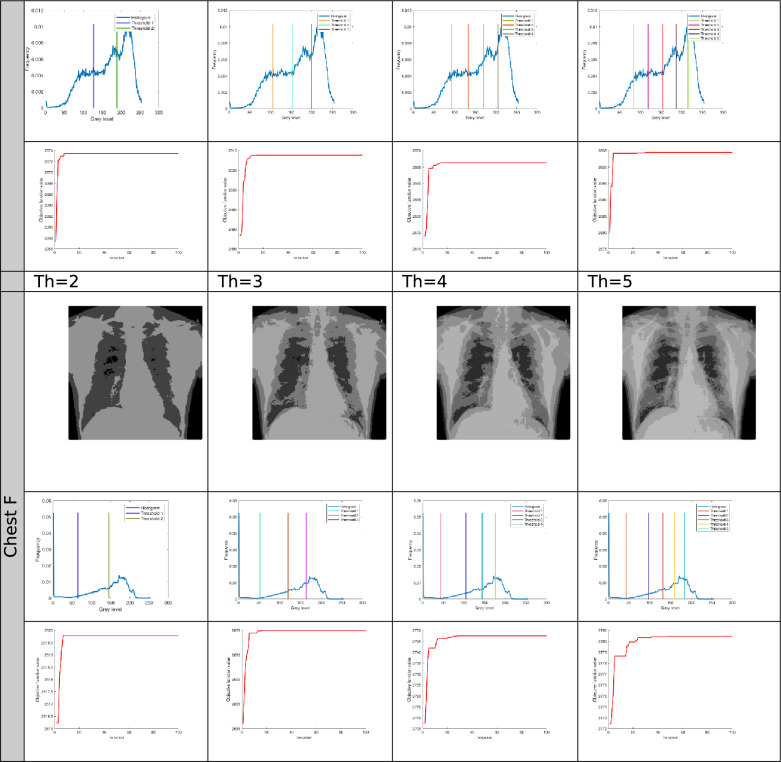
Results after applying ICOOT via Otsu’s method as an objective function over the selected benchmark images.

### Performance evaluation and comparison

The peak signal-to-noise ratio (PSNR) calculates the amount of noise present amid two images (ground truth $${I}_{G}$$ and the segmented image $${I}_{th})$$^72–74^ is calculated as described below.

27 $$PSNR=20 {log}_{10}\frac{255}{RMSE} (dB)$$

28 $$\text{RMSE}=\sqrt{\frac{\sum_{i=1}^{M}\sum_{j=1}^{N}\left({I}_{G}-{I}_{th}\right)}{M X N}}$$

In the context where the image size is denoted as *M × N*, a higher peak signal-to-noise ratio (PSNR) value is preferred, as it signifies a lower amount of noise introduced during processing^75^. The structural similarity index is another parameter used to evaluate the following equation.

29 $$SSIM\left(I,{I}_{s}\right)=\frac{(2{\mu }_{I}{\mu }_{{I}_{S}}+{C}_{1})(2{\sigma }_{I, { I}_{S}}+{C}_{2})}{({\mu }_{I}^{2}+{\mu }_{{I}_{S}}^{2}+{C}_{1})({\sigma }_{I}^{2}+{\sigma }_{{I}_{S}}^{2}+{C}_{2})}$$

where ${\mu }_{{I}_{S}}({\sigma }_{I, { I}_{S}})$ and ${\mu }_{I} \left({\sigma }_{I}\right)$ are the mean intensities of images *I*_*S*_ and *I*, respectively, where ${\sigma }_{I, { I}_{S}}$ represents the governance of *I* and *I*_*S*_, and the ${C}_{1}$ and ${C}_{2}$ coefficient values are equal to 6.5025 and 58.52252, respectively.

The feature similarity index (*FSIM*) is used to define the similarity between two image and its range is [−1, 1]. It can be calculated according to following Eq.

30 $$FSIM=\frac{\sum {S}_{L}{PC}_{m}}{\sum {PC}_{m}}$$

31 $${S}_{L}={S}_{PC}\times {S}_{G}$$

32 $${S}_{pc}=\frac{{2PC}_{1}{PC}_{2}+{T}_{1}}{{PC}_{1}^{2}+{PC}_{2}^{2}+{T}_{1}}$$

33 $${S}_{G}=\frac{{2G}_{1}{G}_{2}+{T}_{2}}{{G}_{1}^{2}+{G}_{2}^{2}+{T}_{2}}$$

where ${PC}_{1}$ and ${PC}_{2}$ are the phase consistency of the original image and segmented image respectively. ${T}_{1}, {T}_{2}$, ${G}_{1}$ and ${G}_{2}$ are positive, and gradient constants of the original and segmented images.

Using six distinct images, an evaluation of the suggested ICOOT for multilevel thresholding segmentation is provided. Table 3 presents the multilevel thresholding results for levels 2, 3, 4, and 5 for seven techniques, such as the AO, WOA, PSO, SSA, QMPA, COOT, AOA, and ICOOT, for the image chest C. Visual evaluation clearly shows that the proposed ICOOT algorithm provides promising results compared with other existing techniques. The acquired results in this table validate the suggested ICOOT’s effectiveness and its capacity to conveniently address the specified task.

**Table 3:**
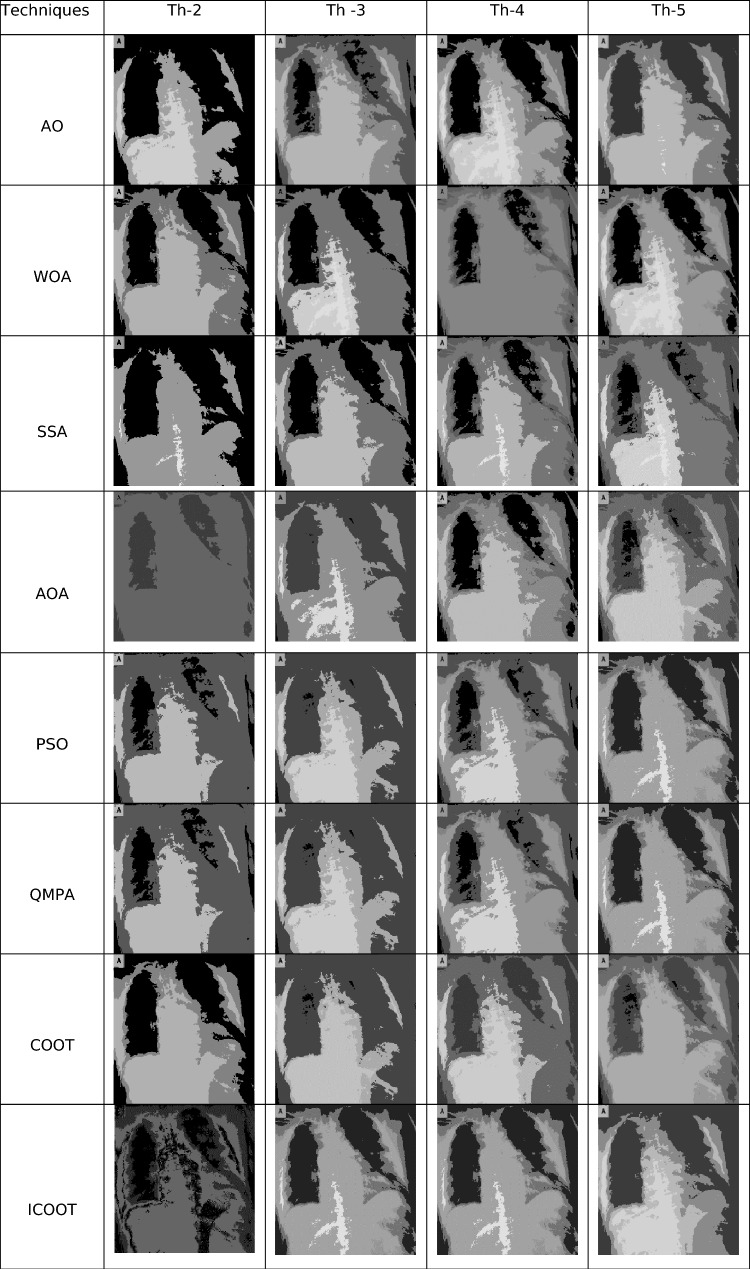
Thresholding results for the ‘Chest C’ image via various techniques.

**Fig. 4:**
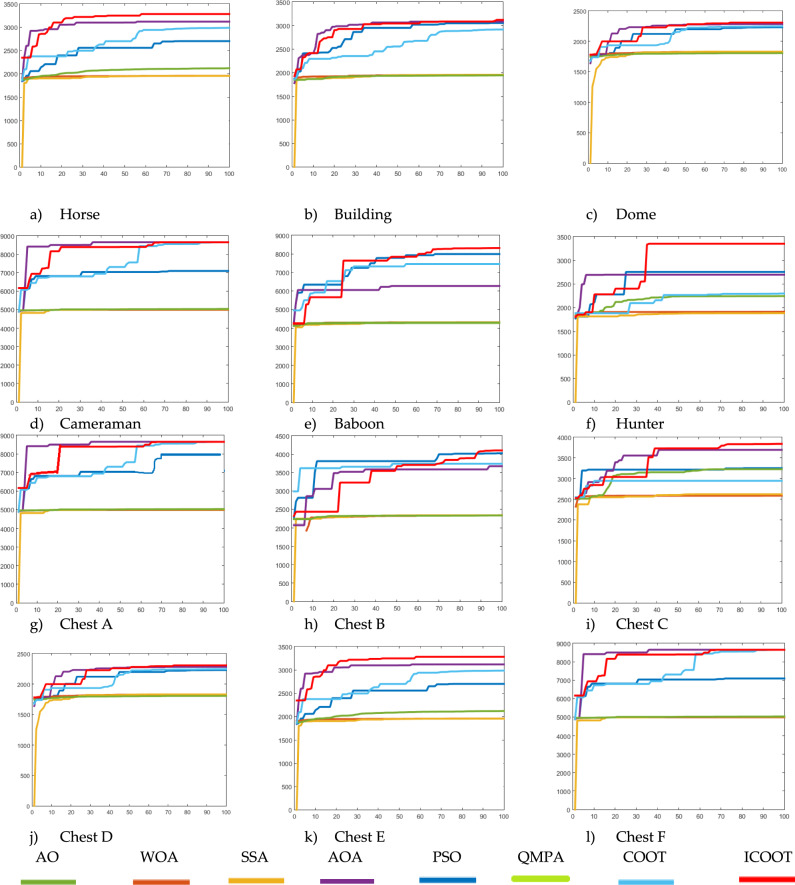
Comparative convergence graph for different test images using state-of-the-art techniques.

In addition to qualitative appearance, quantitative analysis also plays a vital role in result validation. To perform the quantitative analysis, these six images are compared on the basis of the comparative convergence graph in Fig. 4, PSNR in Table 4, SSIM in Table 5, and FSIM in Table 6. The bold values in the following table represent the best results in the row.

**Table 4:** PSNR comparison.

Image	Th	**AO**	**WOA**	**SSA**	**AOA**	**PSO**	**QMPA**	**COOT**	**ICOOT**
Horse	2	11.59619	11.78112	12.43239	12.63767	12.69125	11.82347	11.70256	**13.05978**
	3	**15.307937**	12.97602	14.23469	14.43666	13.40472	14.11783	12.5539	14.66436
	4	14.159587	15.24362	**16.74893**	15.71033	16.64842	15.72964	14.62299	16.36266
	5	15.590393	16.02204	15.55781	**16.81652**	15.8544	15.39825	15.19116	16.18589
Building	2	12.164613	11.54182	12.26758	10.19754	11.71349	**13.64391**	12.49522	12.65703
	3	14.656093	**15.51752**	15.43012	12.98578	14.00204	13.19257	14.47397	14.47253
	4	15.461093	12.20049	15.25485	15.52692	15.03485	15.78642	15.35	**16.84036**
	5	17.379067	16.13135	15.67405	15.29258	16.95624	15.09218	17.22003	**17.7413**
Dome	2	11.71046	13.22549	12.14251	10.87062	8.98892	12.03459	11.81957	**13.41512**
	3	15.38244	13.09156	**18.34402**	14.95033	14.51028	12.52167	15.47871	14.91567
	4	16.02458	14.0508	15.89833	18.01065	14.7851	15.67983	17.02121	**18.55857**
	5	17.836497	17.49149	17.78651	17.05428	17.72475	17.04512	16.80554	**18.40904**
Cameraman	2	12.73669	12.92162	13.57289	13.77817	13.83175	12.96954	12.84306	**14.20028**
	3	**16.448437**	14.11652	15.37519	15.57716	14.54522	15.31542	13.6944	15.80486
	4	15.300087	16.38412	**17.88943**	16.85083	17.78892	16.84319	15.76349	17.50316
	5	16.730893	17.16254	16.69831	**17.95702**	16.9949	16.49288	16.33166	17.32639
Baboon	2	13.305113	12.68232	13.40808	11.33804	12.85399	**14.77305**	13.63572	13.79753
	3	15.796593	**16.65802**	16.57062	14.12628	15.14254	14.27963	15.61447	15.61303
	4	16.601593	13.34099	16.39535	16.66742	16.17535	16.93654	16.4905	**17.98086**
	5	18.519567	17.27185	16.81455	16.43308	18.09674	16.21238	18.36053	**18.8818**
Hunter	2	12.85096	14.36599	13.28301	12.01112	10.12942	13.17596	12.96007	**14.55562**
	3	16.52294	14.23206	**19.48452**	16.09083	15.65078	13.66427	16.61921	16.05617
	4	17.16508	15.1913	17.03883	19.15115	15.9256	16.82413	18.16171	**19.69907**
	5	18.976997	18.63199	18.92701	18.19478	18.86525	18.13692	17.94604	**19.54954**
Chest A	2	0.674371	0.7084	0.68654	0.65767	0.64239	0.75984	0.71795	**0.77548**
	3	0.8276057	0.82327	**0.84588**	0.81247	0.79086	0.71952	0.74828	0.8291
	4	0.810838	0.76667	0.83572	**0.85588**	0.79534	0.85547	0.76403	0.81296
	5	0.8489187	0.85722	0.8246	0.865	0.872	0.87123	0.8077	**0.87373**
Chest B	2	14.146617	12.9197	15.33856	14.59288	13.11365	14.74918	14.29753	**15.59523**
	3	18.80806	17.36488	16.64196	14.29753	16.1011	17.16749	**19.24381**	17.80845
	4	17.022077	16.7795	17.847	15.44791	**17.88083**	16.64825	17.21338	16.91547
	5	18.857707	18.85302	20.47277	20.02741	18.32715	19.81254	18.60749	**20.87642**
Chest C	2	12.481453	13.1436	11.98343	**13.6003**	12.75543	12.43781	12.61301	12.80547
	3	14.60811	12.59469	14.44488	14.91476	15.6324	15.45739	14.558	**15.91476**
	4	17.207307	15.53286	16.34672	**17.42927**	16.44285	14.96872	15.47843	16.66979
	5	16.109907	17.21854	18.34293	17.47001	17.54326	16.50836	**19.54567**	19.21536
Chest D	2	1.586371	1.6204	1.59854	1.56967	1.55439	1.66394	1.62995	**1.68748**
	3	1.7396057	1.73527	**1.75788**	1.72447	1.70286	1.62178	1.66028	1.74115
	4	1.722838	1.67867	1.74772	**1.76788**	1.70734	1.67853	1.67603	1.72496
	5	1.7609187	1.76922	1.7366	1.777	1.784	1.78329	1.7197	**1.78573**
Chest E	2	15.058617	13.8317	16.25056	15.50488	14.02565	15.66138	15.20953	**16.50723**
	3	19.72006	18.27688	17.55396	15.20953	17.0131	18.07951	**20.15581**	18.72045
	4	17.934077	17.6915	18.759	16.35991	**18.79283**	17.55942	18.12538	17.82747
	5	19.769707	19.76502	21.38477	20.93941	19.23915	20.73385	19.51949	**21.78842**
Chest F	2	13.393453	14.0556	12.89543	**14.5123**	13.66743	13.33925	13.52501	13.71747
	3	15.52011	13.50669	15.35688	15.82676	16.5444	16.36842	15.47	**16.82676**
	4	18.119307	16.44486	17.25872	**18.34127**	17.35485	15.87916	16.39043	17.58179
	5	17.021907	18.13054	19.25493	18.38201	18.45526	17.41983	**20.45767**	20.12736

**Table 5:** SSIM Comparison.

Image	**Th**	**AO**	**WOA**	**SSA**	**AOA**	**PSO**	**QMPA**	**COOT**	**ICOOT**
Horse	2	0.18665	0.2017	0.27381	0.23386	**0.28189**	0.19756	0.20966	0.26176
	3	0.35897	0.44463	0.56939	0.50338	0.50338	0.49783	0.54848	**0.58872**
	4	0.35897	0.44463	0.58601	0.50338	0.58872	0.50364	0.54472	**0.59939**
	5	0.48578	0.45064	0.45411	**0.61192**	0.48207	0.38921	0.46297	0.4926
Building	2	0.23631	0.24027	0.2809	0.06324	0.26596	**0.41235**	0.3351	0.28061
	3	0.47014	0.52367	0.37493	0.29417	0.42799	0.29274	0.51006	**0.53337**
	4	0.50939	0.22465	0.51273	0.53004	0.47731	0.55391	0.5055	**0.58868**
	5	0.5179	0.5287	0.51999	0.44699	0.58238	**0.66238**	0.54754	0.62359
Dome	2	0.67437	0.7084	0.68654	0.65767	0.64239	0.75217	0.71795	**0.77548**
	3	0.82761	0.82327	**0.84588**	0.81247	0.79086	0.73248	0.74828	0.8291
	4	0.81084	0.76667	0.83572	0.81468	0.79534	0.84625	0.76403	**0.85588**
	5	0.84892	0.85722	0.8246	0.865	**0.872**	0.86542	0.8077	0.87073
Cameraman	2	0.63925	0.6543	0.72641	0.68646	**0.73449**	0.64193	0.66226	0.71436
	3	0.81157	0.89723	0.93467	0.95598	0.95598	0.95841	0.91557	**0.99945**
	4	0.81157	0.89723	0.91557	0.95598	0.95388	0.94936	0.99732	**0.99989**
	5	0.93838	0.90324	0.90671	**0.94532**	0.93467	0.84218	0.91557	0.94524
Baboon	2	0.68891	**0.86287**	0.7335	0.51584	0.71856	0.86192	0.7877	0.73321
	3	0.92274	0.97627	0.82753	0.74677	0.88059	0.73861	0.96266	**0.98597**
	4	0.96199	0.67725	0.96533	0.98264	0.92991	0.97842	0.9581	**0.99853**
	5	0.9705	0.9813	0.97259	0.89959	0.90525	0.85479	0.96038	**0.99225**
Hunter	2	0.65697	0.691	0.66914	0.64027	0.62499	0.72463	0.70055	**0.75808**
	3	0.81021	0.80587	**0.82848**	0.79507	0.77346	0.71429	0.73088	0.8117
	4	0.79344	0.74927	0.81832	0.79728	0.77794	0.83827	0.74663	**0.83848**
	5	0.83152	0.83982	0.8072	0.8476	**0.8546**	0.84792	0.7903	0.85333
Chest A	2	0.41037	0.4023	0.38729	0.27024	0.40585	0.38416	0.43782	**0.44463**
	3	**0.55431**	0.48971	0.48433	0.5482	0.52659	0.46983	0.46496	0.54374
	4	0.56349	0.49717	0.52804	0.5372	0.54082	0.53876	0.46038	**0.62804**
	5	0.58142	0.65135	0.60801	0.62197	0.67965	0.58694	0.57692	**0.67994**
Chest B	2	0.61362	0.57935	0.62248	0.59898	0.5916	0.61245	0.57949	**0.6381**
	3	**0.72023**	0.6558	0.66776	0.59018	0.62441	0.67193	0.56408	0.67154
	4	0.68623	0.66806	0.67445	0.6605	0.67505	0.66428	**0.68841**	0.67118
	5	0.73833	0.71982	0.71616	0.70312	0.68448	0.69537	0.6851	**0.73876**
Chest C	2	0.52213	0.50284	0.47415	**0.557**	0.51114	0.50987	0.52949	0.55297
	3	0.57947	0.52427	0.5264	0.59575	0.56671	0.54382	0.53508	**0.6069**
	4	0.62103	0.58214	**0.6321**	0.59086	0.55674	0.58729	0.56021	0.62609
	5	0.56706	0.59231	0.62864	0.65383	0.61019	0.60681	0.54948	**0.66026**
Chest D	2	0.66507	0.657	0.64199	0.52494	0.66055	0.64829	0.69252	**0.69933**
	3	**0.80901**	0.74441	0.73903	0.8029	0.78129	0.72315	0.71966	0.79844
	4	0.81819	0.75187	0.78274	0.7919	0.79552	0.79246	0.71508	**0.88274**
	5	0.83612	0.90605	0.86271	0.87667	0.93435	0.84059	0.83162	**0.93464**
Chest E	2	0.86832	0.83405	0.87718	0.85368	0.8463	0.86834	0.83419	**0.8928**
	3	**0.97493**	0.9105	0.92246	0.84488	0.87911	0.92685	0.81878	0.92624
	4	0.94093	0.92276	0.92915	0.9152	0.92975	0.91876	**0.94311**	0.92588
	5	0.99303	0.97452	0.97086	0.95782	0.93918	0.94981	0.9398	**0.99346**
Chest F	2	0.77683	0.75754	0.72885	**0.8117**	0.76584	0.76418	0.78419	0.80767
	3	0.83417	0.77897	0.7811	0.85045	0.82141	0.79853	0.78978	**0.8616**
	4	0.87573	0.83684	**0.8868**	0.84556	0.81144	0.83294	0.81491	0.88079
	5	0.82176	0.84701	0.88334	0.90853	0.86489	0.85261	0.80418	**0.91496**

**Table 6:** FSIM comparison.

**Image**	**Th**	**AO**	**WOA**	**SSA**	**AOA**	**PSO**	**QMPA**	**COOT**	**ICOOT**
Horse	2	0.8577	0.8598	0.8566	0.8329	0.8568	0.8573	0.8583	**0.8843**
	3	0.8147	0.8147	0.8147	0.8141	0.8147	0.8162	0.8144	**0.9310**
	4	0.8466	0.8472	0.8473	0.8361	**0.8476**	0.8468	0.8462	0.8076
	5	0.8862	0.8863	**0.8865**	0.8546	0.8859	0.8851	0.8856	0.8864
Building	2	0.7536	0.7538	0.7536	0.7484	0.7536	0.7543	**0.7544**	0.7005
	3	0.8034	0.8033	0.8008	0.7863	0.8036	0.8042	0.8030	**0.8830**
	4	0.8319	0.8330	0.8315	0.8033	0.8332	0.8325	0.8332	**0.9179**
	5	0.8452	0.8452	0.8452	0.8457	0.8452	0.8467	0.8455	**0.9399**
Dome	2	**0.9244**	0.9238	0.9238	0.9055	0.9235	0.9224	0.9243	0.7572
	3	0.9406	0.9399	0.9394	0.9245	0.9398	0.9398	**0.9409**	0.8458
	4	0.7952	0.7952	0.7952	0.7952	0.7952	0.7967	0.7953	**0.8919**
	5	**0.8735**	0.8734	**0.8735**	0.8725	**0.8735**	0.8728	0.8732	0.5991
Cameraman	2	**0.9377**	0.9373	0.9372	0.9151	0.9372	0.9361	0.9374	0.8429
	3	0.8109	0.8110	0.8109	0.8116	0.8109	0.8123	0.8112	**0.99945**
	4	0.8504	0.8515	0.8500	0.8474	0.8500	0.8492	0.8519	**0.99989**
	5	0.8850	0.8858	0.8846	0.8735	0.8843	0.8831	0.8852	**0.94524**
Baboon	2	0.7244	0.7244	0.7244	0.7241	0.7244	0.7259	0.7244	**0.73321**
	3	0.7810	0.7810	0.7810	0.7796	0.7810	0.7804	0.7808	**0.98597**
	4	0.8235	0.8233	0.8236	0.8097	0.8233	0.8226	0.8235	**0.99853**
	5	0.8578	0.8579	0.8575	0.8250	0.8575	0.8584	0.8579	**0.99225**
Hunter	2	0.8248	0.8288	0.8287	0.8201	0.8288	0.8297	0.8284	**0.95808**
	3	0.8832	0.8831	0.8831	0.8622	**0.8852**	0.8845	0.8825	0.8117
	4	0.9128	0.9117	0.9115	0.8893	0.9109	0.9104	**0.9129**	0.83848
	5	0.7573	0.7573	0.7573	0.7575	0.7573	0.7588	0.7574	**0.85333**
Chest A	2	0.8913	0.8937	0.8930	0.8419	0.8936	0.8947	0.8937	**0.8987**
	3	0.9265	0.9279	0.9266	0.8839	0.9281	0.9292	0.9265	**0.94374**
	4	0.7662	0.7662	0.7662	**0.7664**	0.7662	0.7655	0.7662	0.62804
	5	0.8335	0.8342	0.8335	0.8258	0.8335	0.8346	0.8351	**0.97994**
Chest B	2	0.9150	0.9156	0.9155	0.8914	0.9154	0.9158	0.9156	**0.9381**
	3	0.7291	0.7292	0.7291	**0.7296**	0.7291	0.7204	0.7295	0.67154
	4	**0.8029**	0.8028	0.8026	0.7895	**0.8029**	0.8002	0.8022	0.67118
	5	0.8503	0.8498	0.8472	0.8231	0.8498	0.8509	0.8501	**0.97386**
Chest C	2	0.9128	0.9117	0.9115	0.8893	0.9109	0.9123	0.9129	**0.95297**
	3	0.8066	0.8066	0.8065	0.8074	0.8066	0.8078	0.8066	**0.8869**
	4	0.8307	0.8309	0.8312	0.8245	0.8312	0.8326	0.8303	**0.90609**
	5	0.8579	0.8582	0.8583	0.8412	0.8575	0.8594	0.8575	**0.94026**
Chest D	2	0.7218	0.7220	0.7218	0.7165	0.7218	0.7203	**0.7224**	0.69933
	3	0.7648	0.7648	0.7633	0.7512	0.7647	0.7659	0.7647	**0.79844**
	4	0.7890	0.7933	0.7870	0.7714	0.7897	0.7985	0.7913	**0.88274**
	5	0.7886	0.7886	0.7886	0.7885	0.7886	0.7874	0.7887	**0.93464**
Chest E	2	0.9137	0.9131	0.9127	0.8849	0.9123	0.9133	**0.9138**	0.8928
	3	0.9344	**0.9352**	0.9325	0.9138	0.9328	0.9351	0.9340	0.92624
	4	0.7584	0.7584	0.7584	0.7579	0.7584	0.7596	0.7582	**0.92588**
	5	0.8466	0.8471	0.8465	0.8468	0.8465	0.8473	0.8475	**0.99346**
Chest F	2	**0.9223**	**0.9223**	0.9220	0.9000	0.9222	0.9231	0.9216	0.80767
	3	0.8392	0.8393	0.8392	0.8395	0.8392	0.8407	0.8395	**0.8616**
	4	0.8902	0.8919	0.8894	0.8864	0.8894	0.8895	**0.8926**	0.88079
	5	0.9107	0.9112	0.9104	0.9060	0.9101	0.9114	0.9112	**0.91496**

In Fig. 4, comparative conversion graphs are plotted for a threshold value of 5. The provided graphs show that, compared with the other techniques, the ICOOT algorithm achieves better performance with quicker convergence and more consistent precision. Practically all the test images show remarkable smoothing behavior in the convergence curves. Furthermore, the suggested technique’s curve convergence graph is consistent, demonstrating that the proposed ICOOT’s capacity to prevent the difficulty of local optima is consistent. Finally, compared with the previous comparison approaches, the suggested ICOOT yields the best results in all of the investigated scenarios, i.e., in all twelve images.

In Table 4, the PSNR results for proposed ICOOT algorithm and seven benchmark metaheuristic algorithms (AO, WOA, SSA, AOA, PSO, QMPA, and COOT) across a twelve different test images from both natural and medical domains, with different threshold levels (Th = 2, 3, 4, 5) are presented. AO achieves the highest PSNR value three times, the WOA two times, and SSA achieves better results in six different cases. At higher thresholds, ICOOT maintains a competitive edge, ICOOT records 20.8764 at Th = 5, the highest among all methods for chest B. The AOA yields the best results eight times, and the PSO achieves the best results only two times. The QMPA and COOT won in two and four cases, respectively. The proposed ICOOT achieves the best PSNR results twenty-two times, indicating superior segmentation quality with minimal distortion relative to the original image. The WOA and QMPA are both on the least side and provide the minimum PSNR value, i.e., only two out of forty-eight total experiments. ICOOT’s superiority is most pronounced in high-texture images (Baboon) and high-detail medical scans (Chest B, Chest E), where accurate threshold selection is critical. In a few cases (e.g., Horse at Th = 4), other algorithms perform slightly better, which could be due to dataset-specific histogram distributions.

Table 5 reports the Structural Similarity Index Measure (SSIM) values for a total of forty-eight experiments for the ICOOT and other seven benchmark metaheuristic algorithms (AO, WOA, SSA, AOA, PSO, QMPA, COOT) across multiple images and threshold levels (Th = 2, 3, 4, 5). SSIM reflects perceptual image quality, measuring how well structural information and contrast are preserved after segmentation. ICOOT reaches the highest SSIM at Th = 3 (0.5887) and Th = 4 (0.5994), outperforming the next best method (SSA, 0.5860) by a noticeable margin, indicating better edge and texture preservation for horse image. ICOOT records a leading value of 0.6236 at Th = 5 for Building image, surpassing the closest competitor (QMPA, 0.6569 at Th = 5) in maintaining overall structural similarity.

The AO achieves the highest SSIM value four times, the WOA one time, and the SSA achieves better results in four different cases. The AOA and PSO achieve the best results only four times. The QMPA and COOT yield the best results two times in each case. The proposed ICOOT achieves the best SSIM results twenty-seven times. The WOA is on the least side and provides the minimum SSIM value, i.e., only one out of forty-eight total experiments.

ICOOT’s advantage is more pronounced at higher thresholds (Th = 4 and Th = 5), where preserving detail while segmenting becomes more challenging. For texture-rich images (Baboon) and fine-detail medical images (Chest E), ICOOT achieves near-perfect SSIM scores, confirming its strong balance between segmentation sharpness and artifact minimization. Slightly lower SSIM in some low-threshold cases suggests that the benefits of ICOOT are more evident when multiple thresholds are used, leveraging its improved exploration and exploitation capabilities. The SSIM results further validate that ICOOT outperforms traditional and contemporary metaheuristic methods in maintaining the structural and perceptual integrity of segmented images across diverse datasets and complexity levels.

Table 6 presents the Feature Similarity Index Measure (FSIM) values for the proposed ICOOT algorithm and seven benchmark metaheuristic algorithms (AO, WOA, SSA, AOA, PSO, QMPA, COOT) across different images and threshold levels (Th = 2, 3, 4, 5). FSIM evaluates image quality based on low-level features such as phase congruency and gradient magnitude, which are important for human visual perception. Higher FSIM values indicate better preservation of structural and perceptual features during segmentation. The AO achieves the highest FSIM value five times, and the WOA, SSA, and AOA achieve the best result values in only two different cases. PSO achieves the best values four times in a total of forty-eight cases. The QMPA and COOT won in one and six cases, respectively. The proposed ICOOT achieves the best FSIM results thirty-one times. The WOA, SSA, and AOA are on the lowest side and provide the best value only once the FSIM in forty-eight experiments.

In several cases (Horse, Baboon, Building), FSIM for ICOOT is higher than all other algorithms, reflecting better feature-level segmentation quality. Unlike PSNR or SSIM where differences are sometimes marginal, FSIM clearly shows ICOOT’s ability to maintain phase congruency and gradient information even in challenging images. Performance gains are especially evident in high-threshold scenarios, suggesting that ICOOT’s improved exploration–exploitation mechanism allows for more optimal feature boundary detection. The FSIM analysis reinforces the findings from PSNR and SSIM, demonstrating that ICOOT consistently preserves critical image features, edges, and textures across diverse datasets and segmentation complexities, making it a robust choice for both natural and medical image segmentation tasks.

In terms of the PSNR and SSIM, the suggested ICOOT produced new positive findings in nearly all of the test situations. The acquired results in the following tables verify the suggested ICOOT functionality and its capacity to successfully address the specified tasks. The accompanying findings demonstrate and validate the suggested algorithm’s excellent capacity to solve thresholding issues, and its goal is to find reliable solutions, i.e., optimal threshold values, in this sector.

Table 7 presents the comparison of multilevel threshold values obtained for each test image using the proposed ICOOT algorithm and the comparative methods (AO, WOA, SSA, AOA, PSO, QMPA, and COOT). The thresholds were computed for four different levels (thresh = 2, 3, 4, 5), corresponding to increasing segmentation granularity. From the table, it can be observed that ICOOT consistently produces threshold sets that are more balanced and evenly distributed across the intensity range compared to the other algorithms. This distribution is particularly evident in images with high texture complexity such as Baboon and Hunter, where ICOOT avoids clustering thresholds in narrow intensity regions — a common limitation in several baseline algorithms. In medical images (Chest A–F), the proposed method generates threshold values that more accurately correspond to the underlying tissue intensity variations, aiding in clearer separation between infected and normal regions. For example, in Chest E with k = 5, ICOOT’s thresholds are more uniformly spaced, which enhances the delineation of pathological structures, as confirmed by the corresponding PSNR and SSIM results.

**Table 7:** Threshold value comparison.

Image name	Th	AO	WO	SS	AOA	PSO	QMPA	COOT	ICOOT
Baboon	2	103 150	106 153	98 145	102 149	99 146	97 144	104 151	101 148
	3	90 126 160	93 129 163	85 121 155	89 125 159	86 122 156	84 120 154	91 127 161	88 124 158
	4	75 108 135 165	78 111 138 168	70 103 130 160	74 107 134 164	71 104 131 161	69 102 129 159	76 109 136 166	73 106 133 163
	5	72 103 129 150 173	75 106 132 153 176	67 98 124 145 168	71 102 128 149 172	68 99 125 146 169	66 97 123 144 167	73 104 130 151 174	70 101 127 148 171
Horse	2	113 163	116 168	108 160	112 164	109 161	107 159	114 166	111, 161
	3	103 157 207	108 162 212	98 152 202	102 156 206	99 153 203	97 151 201	104 158 208	101 155 205
	4	87 129 163 202	90 132 166 205	82 124 158 197	86 128 162 201	83 125 159 198	81 123 157 196	88 130 164 203	85 127 161 200
	5	89 120 152 179 214	92 123 155 182 217	85 116 148 175 210	88 119 151 178 213	85 116 148 175 210	83 114 146 173 208	90 121 153 180 215	87 118 150 177 212
Building	2	97 157	100 160	94 154	96 156	95 155	93 153	98 158	95 155
	3	89 141 198	92 143 200	86 135 192	88 139 196	87 136 193	85 134 191	90 143 200	87 138 195
	4	79 125 170 227	82 128 173 230	76 122 167 224	78 124 169 226	77 121 166 223	75 119 164 221	80 126 171 228	77 123 168 225
	5	69 102 140 163 220	72 105 143 166 223	66 99 137 160 217	68 101 139 162 219	67 100 138 161 218	65 98 136 159 216	70 103 141 164 221	67 100 138 161 218
Dome	2	74 147	77 150	69 142	73 146	72 145	70 143	75 148	72 145
	3	77 114 165	80 117 168	72 109 160	76 113 164	75 112 163	73 110 161	78 115 166	75 112 163
	4	77 112 147 177	80 115 150 180	72 107 142 172	76 111 146 176	75 110 145 175	73 108 143 173	78 113 148 178	75 110 145 175
	5	74 107 147 187 259	77 110 150 190 262	69 104 142 182 254	73 106 146 186 258	72 105 145 185 257	70 103 143 183 255	75 110 148 188 260	72 105 145 185 257
Camera man	2	72 146	75 149	69 141	71 145	70 144	68 142	73 147	70 144
	3	60 120 157	63 123 160	56 115 152	59 119 156	58 118 155	56 116 153	61 121 158	58 118 155
	4	44 97 142 172	47 100 145 175	40 93 138 168	43 96 141 171	42 95 140 170	40 93 138 168	45 98 143 173	42 95 140 170
	5	39 84 124 151 175	42 87 127 154 178	35 80 120 147 171	38 83 123 150 174	37 82 122 149 173	35 80 120 147 171	40 85 125 152 176	37 82 122 149 173
Hunter	2	53 118	56 121	48 113	52 117	49 116	47 114	54 121	51 116
	3	39 88 135	42 91 138	33 82 129	37 86 133	34 83 130	32 81 128	40 89 136	36 85 132
	4	28 64 103 142	31 67 106 145	23 59 98 137	26 62 101 140	24 60 99 138	22 58 97 136	29 65 104 143	26 62 101 140
	5	23 53 87 120 149	26 56 90 123 152	18 48 82 115 144	21 51 85 118 147	19 49 83 116 145	17 47 81 114 143	24 54 88 121 150	21 51 85 118 147
Chest A	2	50 102	53 105	47 99	49 101	48 100	46 98	51 103	48 100
	3	47 89 140	50 92 143	44 83 134	46 88 139	45 86 137	43 84 135	48 95 146	45 86 137
	4	38 75 126 151	41 78 129 154	35 70 121 146	37 74 125 150	36 73 124 149	34 71 122 147	39 76 127 152	36 73 124 149
	5	26 54 94 120 145	29 57 97 123 148	23 51 91 117 142	25 53 93 119 144	24 52 92 118 143	22 50 90 116 141	27 55 95 121 146	24 52 92 118 143
Chest B	2	90 151	93 154	85 146	89 150	88 149	86 147	91 152	88 149
	3	43 99 155	46 102 158	35 91 147	42 98 154	38 94 150	36 92 148	44 100 156	38 94 150
	4	44 82 139 183	47 85 142 186	36 74 131 175	43 81 138 182	39 77 134 178	37 75 132 176	45 83 140 184	39 77 134 178
	5	43 71 116 152 166	46 74 119 155 169	35 63 108 144 158	42 70 115 151 165	37 67 112 148 162	35 65 110 146 160	44 72 117 153 167	41 69 114 150 164
Chest C	2	62 144	61 143	60 145	62 146	63 145	61 144	62 145	61 144
	3	40 90 145	37 91 148	39 92 146	38 90 148	39 90 146	38 90 148	39 92 147	38 91 147
	4	41 86 138 167	40 83 136 165	41 84 139 166	42 85 137 165	40 85 138 167	42 84 136 165	41 83 139 166	41 84 137 166
	5	31 64 96 147 172	32 62 94 145 170	31 63 95 147 172	30 62 94 147 171	31 64 94 146 172	32 63 94 145 170	30 62 94 146 171	31 63 94 146 171
Chest D	2	102 164	105 167	97 159	101 163	100 162	98 160	102 164	100 162
	3	58 167 192	61 170 195	54 161 186	57 164 189	55 161 186	53 159 184	58 167 192	57 164 189
	4	44 108 151 184	47 111 154 187	40 104 147 180	43 107 150 183	41 105 148 181	39 103 146 179	44 108 151 184	43 107 150 183
	5	42 93 130 179 201	45 96 133 182 204	38 89 126 175 197	41 92 129 178 200	39 90 127 176 198	37 88 125 174 196	42 93 130 179 201	41 92 129 178 200
Chest E	2	132 191	135 194	127 186	131 190	130 189	128 187	131 190	130 189
	3	112 159 202	115 162 205	107 154 197	111 158 201	110 157 200	108 155 198	111 158 201	110 157 200
	4	91 145 173 215	94 148 176 218	86 140 168 210	90 144 172 214	89 143 171 213	87 141 169 211	90 144 172 214	89 143 171 213
	5	96 126 152 188 231	99 129 155 191 234	91 121 147 183 226	95 125 151 187 230	92 122 148 184 227	90 120 146 182 225	95 125 151 187 230	94 124 150 186 229
Chest F	2	64 148	67 151	61 143	63 147	62 146	60 144	61 143	62 146
	3	55 133 164	58 136 167	52 128 159	54 131 162	53 131 162	51 129 160	52 128 159	53 131 162
	4	44 115 143 181	47 118 146 184	41 110 138 176	43 113 141 179	42 113 141 179	40 111 139 177	41 110 138 176	42 113 141 179
	5	43 102 139 171 190	46 105 142 174 193	40 97 134 166 185	42 101 138 170 189	41 100 137 169 188	39 98 135 167 186	40 97 134 166 185	41 100 137 169 188

The consistency of ICOOT across different image categories (natural scenes and medical scans) highlights its adaptability to varying histogram distributions, from unimodal to highly multimodal. Furthermore, the stability of threshold patterns across multiple runs suggests robust convergence behavior with minimal sensitivity to initial population randomness. Overall, this threshold-level comparison complements the performance metrics (PSNR, SSIM, FSIM) by demonstrating that the improved segmentation quality is inherently linked to the algorithm’s capability to identify optimal intensity partitions that preserve both structural detail and perceptual quality.

## Conclusion

Multilevel thresholding remains a critical aspect of image segmentation, but its computational complexity increases with the number of thresholds. This work introduces an improved version of the COOT optimization algorithm, named ICOOT, which incorporates quasi opposition-based Learning (QOBL) and Lévy flights to enhance the balance between exploration and exploitation, refining the updated locations of the search agents. It has been applied to segment digital and CT images at multiple levels (i.e., 2, 3, 4, and 5). The ICOOT algorithm has demonstrated superior performance in segmenting both natural and CT images, outperforming contemporary optimizers such as the AO, WOA, SSA, AOA, PSO, and the marine predator algorithm in terms of the PSNR, SSIM, and FSIM metrics. Moreover, both quantitative and qualitative studies demonstrate better results than other algorithms do. ICOOT produces high-quality segmented images across various threshold levels. The potential for ICOOT application can be expanded to other image processing tasks, such as edge detection and noise reduction, as well as real-time applications, such as live video processing and autonomous driving systems. Future research could explore hybrid approaches that combine ICOOT with other optimization or machine learning methods, automated parameter tuning for different image types, scalability for high-resolution images through parallelization, and domain-specific adaptations for medical imaging, satellite imagery, and industrial inspection.

## Data Availability

The data are publicly available at https://www2.eecs.berkeley.edu/Research/Projects/CS/vision/bsds/.
